# Attitudes of General Practitioners Toward Prescription of Mobile Health Apps: Qualitative Study

**DOI:** 10.2196/21795

**Published:** 2021-03-04

**Authors:** Aline Sarradon-Eck, Tiphanie Bouchez, Lola Auroy, Matthieu Schuers, David Darmon

**Affiliations:** 1 Aix Marseille University, INSERM, IRD, SESSTIM Marseille France; 2 Institut Paoli-Calmettes CanBios UMR1252 Marseille France; 3 Université Côte d’Azur, Rétines, Healthy, DERMG Nice France; 4 Université Grenoble Alpes, Centre National de la Recherche Scientifique Sciences Po Grenoble, Pacte Grenoble France; 5 Department of General Medicine Rouen University Hospital Rouen France; 6 Department of Biomedical Informatics Rouen University Hospital Rouen France; 7 INSERM, U1142, Laboratoire d’Informatique Médicale et d’Ingénierie des Connaissances en e-Santé (LIMICS) Sorbonne Université Paris France

**Keywords:** mobile applications, qualitative research, general practitioners, France, mobile phone

## Abstract

**Background:**

Mobile health (mHealth) apps are a potential means of empowering patients, especially in the case of multimorbidity, which complicates patients’ care needs. Previous studies have shown that general practitioners (GPs) have both expectations and concerns regarding patients’ use of mHealth apps that could impact their willingness to recommend the apps to patients.

**Objective:**

The aim of this qualitative study is to investigate French GPs’ attitudes toward the prescription of mHealth apps or devices aimed toward patients by analyzing GPs’ perceptions and expectations of mHealth technologies.

**Methods:**

A total of 36 GPs were interviewed individually (n=20) or in a discussion group (n=16). All participants were in private practice. A qualitative analysis of each interview and focus group was conducted using grounded theory analysis.

**Results:**

Considering the value assigned to mHealth apps by participants and their willingness or resistance to prescribe them, 3 groups were defined based on the attitudes or positions adopted by GPs: *digital engagement* (favorable attitude; mHealth apps are perceived as additional resources and complementary tools that facilitate the medical work, the follow-up care, and the monitoring of patients; and apps increase patients’ compliance and empowerment); *patient protection* (related to the management of patient care and fear of risks for patients, concerns about patient data privacy and security, doubt about the usefulness for empowering patients, standardization of the medical decision process, overmedicalization, risks for individual freedom, and increasing social inequalities in health); *doctor protection* (fear of additional tasks and burden, doubt about the actionability of patient-gathered health data, risk for medical liability, dehumanization of the patient-doctor relationship, fear of increased drug prescription, and commodification of patient data).

**Conclusions:**

A deep understanding of both the expectations and fears of GPs is essential to motivate them to recommend mHealth apps to their patients. The results of this study show the need to provide appropriate education and training to enhance GPs’ digital skills. Certification of the apps by an independent authority should be encouraged to reassure physicians about ethical and data security issues. Our results highlight the need to overcome technical issues such as interoperability between data collection and medical records to limit the disruption of medical work because of data flow.

## Introduction

### Background

Mobile health (mHealth) technologies are apps or devices designed to deliver health information, collect clinical health data, provide real-time monitoring of patients’ vital signs, and sometimes direct therapeutic interventions. The medical and health care literature presents mHealth apps as a means of empowering patients by providing them with medical and health-related information and education, improving their compliance with treatment, and helping them manage their own health [1]. Many patients consider that mHealth apps aimed toward enabling patients to facilitate the self-management of their illnesses [2], especially in the case of multimorbidity that complexifies patients’ care needs, leading to difficulties in coping with illnesses and an increased burden of illness and treatment [3]. In addition, several clinical trials have shown a meaningful effect on health outcomes attributable to apps that mostly address diabetes, mental health, and obesity [4].

In France, as in most European countries, general practitioners (GPs) occupy a central place in the health care system. In 2014, more than 80% of French people aged 15 years and older had consulted a GP during the year [5]. Not only are GPs primary care providers and gatekeepers but they also manage and coordinate the health care pathways of their patients [6-8].

Multimorbidity, defined as the simultaneous coexistence of more than 1 chronic condition in a single individual, is increasing in primary care and leading to more complex medical care [9]. GPs play a central role in organizing care delivery for patients with multimorbidity [10] and managing complex decision making and providing patients with support to self-manage their illnesses [11]. To provide a comparison with another European country, in Scotland, about a quarter (23.2%) of the population has chronic multimorbidities [12]. The increase in multimorbidity does not only concern the older adults; by the age of 50 years, half of the Scottish patients had at least one morbidity in 2007 [12]. This leads us to believe that a large proportion of chronically ill patients should be concerned with effective tools or devices that could help both patients and their physicians to manage their illnesses, especially through mHealth technologies.

mHealth apps are available on smartphones or tablets. In 2019, almost 8 out of 10 French people had a smartphone, but only 44% of those aged 70 years and older had a smartphone [13]. In this population, the use of a smartphone to browse the internet decreases after 40 years of age: 85% (40-59 years), 69% (60-69 years), and 57% (70 years and older). In the end, only 26% of all people aged 70 years and older browse the internet on a mobile device; however, this rate has been increasing over the past year (+7 points for those aged 70 years and older and +10 points for those aged 60-69 years) [13].

In France, GPs can prescribe some connected devices, that is, these devices are reimbursed by the health insurance scheme (eg, glucometers such as Freestyle Libre). Currently, only 1 app (Moovcare) is reimbursed by the health insurance scheme and can be prescribed for the clinical follow-up of patients with lung cancer. Other stand-alone mHealth apps are not yet reimbursed; therefore, doctors can only advise them. However, will French GPs be ready to prescribe mHealth apps to their patients when these apps will be certified by health authorities?

Both a Swedish [14] and an Australian [15] study suggest that physicians tend to recommend health apps to their patients sparingly, even if they have a positive attitude and perceive an improvement in patients’ self-management ability as the main benefit of health apps [14]. The lack of knowledge of effective apps seems to be the main barrier [15,16]. Dutch GPs seem to have a supporting or mixed attitude toward the integration of mHealth in primary care [17]. A German survey [18] conducted with 1070 GPs showed that “skeptical physicians”—who are concerned about data security, reliability of apps, legal questions, and additional burdens—are more numerous (44%) than the “open-minded physicians” (35%), who emphasized motivational and compliance advantages. However, a majority of the GPs interviewed perceived valuable application potential for health apps and positive contributions to health care and recovery. They are now in favor of recommending mHealth apps to patients, even if they had been reluctant to do so in the past.

### Objectives

The aim of this study is to investigate French GPs’ attitudes toward prescription or recommendation of mHealth apps or devices aimed toward patients by analyzing their perceptions and expectations of mHealth technologies. On the basis of a sociological approach and following previous research [19-21], this paper aims to provide an empirically grounded typology of the attitudes of French GPs toward the prescription of mHealth apps.

## Methods

### Design

This qualitative study, which was conducted from October 2018 to March 2019, involved semistructured face-to-face interviews and 2 focus groups (FGs) with GPs.

Ethical approval was obtained from the French Institute of Medical and Health Research Ethics Committee (IORG0003254 and FWA00005831) and the Institutional Review Board (IRB00003888; opinion number 18-499). All participants gave their consent after receiving both oral and written information about the study.

### Study Participants and Recruitment

As French general practice is predominantly in the private sector [22], all participants were in private practice.

On the basis of a purposive sample strategy, 20 GPs working in South-East France were interviewed individually. To obtain various viewpoints about GPs’ perceptions and expectations of mHealth apps aimed toward patients, we interviewed GPs who were at different stages in their career trajectory, worked in areas with different population densities, and worked in solo or group practice (single specialty or multispecialties; Table 1). As part of the recruitment of study participants, 90 physicians were initially contacted using a professional phone directory. Of these, 12 agreed to participate in an interview, 6 refused to participate, and a large majority (72/90, 80%) did not give an answer (positive or negative). A total of 8 participants were recruited via snowball sampling. The recruitment process ended when the theoretical saturation point (defined later) was reached.

**Table 1:** General practitioners’ characteristics.

Characteristics		Individual interviews (n=20)	Focus groups (n=16)	Total sample (N=36)
**Age (years), n (%)**				
	<40	5 (25)	9 (56)	14 (39)
	40-50	1 (5)	1 (6)	2 (6)
	50-60	6 (30)	4 (25)	10 (28)
	>60	8 (40)	2 (13)	10 (28)
**Gender, n (%)**				
	Male	12 (60)	8 (50)	20 (56)
	Female	8 (40)	8 (50)	16 (45)
**Private practice context, n (%)**				
	Solo practice	5 (25)	1 (6)	6 (17)
	Group practice	12 (60)	9 (56)	21 (28)
	Multidisciplinary primary health care organization	3 (15)	6 (38)	9 (25)
**Involved in medical training of residents, n (%)**				
	Yes	14 (70)	16 (100)	30 (83)
	No	6 (30)	0 (0)	6 (17)

In addition, to debate about physicians’ expectations for mHealth apps, 2 FGs were organized with 7 and 9 GPs each. All 16 participants were involved in university training for the GP residents. The first FG was conducted in Nice (South-East of France), whereas the second was held in Rouen (North-West of France; Table 1).

### Procedure

Individual interviews were conducted by a medical sociologist (LA). She approached the participants by email and conducted face-to-face interviews at physicians’ offices with willing participants for a duration of 28 to 87 minutes (median=40).

The FGs were organized in a university setting. Each FG was conducted by an experienced moderator (TB and MS), and the cumulative duration of the interviews was 132 minutes.

An interview guide was used to conduct the interviews and FG discussions. It covered the following areas: knowledge and interest in digital technologies, possible use of mHealth apps, experience of recommending mHealth apps or devices, effects on doctor-patient relationship, risks and benefits for the patient, doubts and fears, and effects on patient responsibility and autonomy. We used a FG approach designed to explore the stable opinions, norms, and group processes that arise within the group.

Interviews and group discussions were audio recorded and transcribed. All personally identifiable information was removed from the transcripts. Interviews and FG were identified with a code: E (for interview)+number, FG (for focus group)+city initial (R for Rouen, N for Nice)+age.

### Data Analysis

A qualitative analysis of each interview and FG was conducted using grounded theory coding analysis [23]. Transcripts of the personal and collective interviews were hand coded using an iterative inductive process. The first step was initial coding, and segments of transcripts were coded into categories until the categories accounted for all the variations in the data. In the second step (focused coding), selective coding and relationships between the categories were refined and core categories were identified and arranged into broad emergent thematic categories. The third step (axial coding) aimed at identifying the core phenomenon, causal conditions, resultant strategies, context, and consequences [23].

To propose a researcher-constructed typology to simplify and “to reduce the complexity of the natural world by focusing attention on a usually small number of elements or issues of interest to the researcher” [24], we searched for an empirical dimension connected with our research object (GPs’ perceptions of mHealth, expectations, concerns, and fears). We identified the dimension of the value given by GPs to the mHealth apps aimed toward patients. This empirical dimension has emerged as a central issue in our data and allowed to organize the data in new ways following 2 axes: (1) the value of the apps for patients (from high to low) and (2) the value of the apps for medical work (from high to low). Next, we reassembled the data into 4 themes (Table S1 given in Multimedia Appendix 1): (1) GPs’ willingness: apps as additional resources to medical work, (2) GPs’ skepticism or resistance, (3) high value of the apps for patient, and (4) less value of the apps and risks for patient.

Finally, we constructed a typology that is a theorizing process to understand and explain the phenomenon at hand, that is, the range of attitudes of GPs toward the possible prescription of mHealth apps. Considering the position of GPs’ concerns and expectations on the value axes, 3 groups of attitudes or positions adopted by the GPs were identified and defined: *digital engagement*, *patient protection*, and *doctor protection*.

Interview transcripts were coded by LA and checked by AS. The FG transcripts were coded by TB and checked by DD and AS. To ensure the validity of the analysis, the following strategies were used: saturation and constant comparison. Data collection and analysis were conducted simultaneously, leading the purposeful sampling to confront the validity of analysis or to explore new concepts until no further new items emerged (theoretical concept saturation was reached). The data codes, categories, and themes were constantly checked, compared, and contrasted. Selective coding and relationships between the categories were discussed within our interdisciplinary research team that included a medical anthropologist (AS), a medical sociologist (LA), and 3 GPs (DD, TB, and MS) who are involved in research in general practice and training of junior GPs. These findings were also examined in relation to the existing literature to identify any inaccuracies and/or misinterpretations.

## Results

### Attributes and Types

Considering the value assigned to mHealth apps by participants and their willingness or resistance to prescribe, we identified 3 positions: *digital engagement*, *patient protection*, and *doctor protection*. However, these categories are ideal types of attitudes and not of people. In other words, each GP interviewed tended to adopt one type of attitude but had an ambivalent discourse regarding specific aspects of the prescription of mHealth apps or the patients’ use of apps.

We detail these 3 positions by giving their characteristics, which we illustrate with a few short relevant verbatims. In addition, the reader may find other illustrative excerpts from interviews classified by themes during the final coding in Table S1 in Multimedia Appendix 1.

### Digital Engagement

Some of the GPs adopted a rather favorable posture for mHealth and perceived mHealth apps as additional resources. Most of them were familiar with the tools; they used professional apps and had tested some mHealth apps:

One that is very often recommended for chronic lumbago is the application Activ’Dos. I know that one well because I have used it myself. It is rather well done. (...) I tested it on myself, so I advise it for my patients and show them how to use it.

E19

Although our sample is not a representative one, it is instructive to stress that among the 20 GPs interviewed individually, 10 used professional apps, 11 knew of one or more mHealth apps aimed toward patients, and 6 had previously advised an mHealth app to their patients. Most of them reported a lack of knowledge or familiarity with these tools and even a lack of interest. However, they expressed a desire to gain more digital skills.

The main perceived benefit was the facilitation of medical work and follow-up care. Participant GPs stressed the reliability and objectivity of measurements allowed by the app, its traceability, and the possibility of a history of measurements that facilitate medical work in terms of diagnosis and patient care plan:

[About blood pressure measurements] An application would be much better. I give them papers with charts to fill in, then they bring me their charts...which is just fine! I scan them and put them in the files, but if I had that on the Internet, it would be...much simpler.

E15

A patient’s digital logbook is perceived to be more convenient than the usual paper logbook. From this point of view, digital tools are additional control devices that could facilitate better monitoring of patients, because this self-monitoring could be more intensive and occur in real time:

It should be a tool that facilitates the transmission of information from him to me. (...) It would be a bit like having the nurse visit the patient every day (...) with an eye on the patient’s state of health on a daily basis. I think that would be useful.

E10

The case of diabetic patients was often cited as an example of the utility of the mHealth app, especially to help physicians in remote decision making. On the basis of the history of blood glucose tests transmitted via an app connected to the medical records, and without meeting the patient, the physician could adjust the therapeutic regimen and dosages:

The major interest is the communication of data (...) so the interest is double for the diabetic, (...) there is no need to go anywhere. All of the data are transferred to the doctor. He has, for example, a week of (blood sugar) curves and can remotely (...) adapt the insulin and the dosage and the therapeutic measures for the diabetic.

E7

From their point of view, the mHealth app could serve as an extension supporting previously practiced medical work for tasks such as relaying advice related to healthy food or physical activities, self-monitoring, and education. GPs interviewed presented mHealth apps aimed toward patients as complementary tools to medical practice; however, they stressed that an app should not replace the physician:

[mHealth apps] This would be to help the doctor on a daily basis, or to help the patient in the care and management of his illness, but certainly not to make medical diagnoses or to provide pseudodiagnoses (...) in the place of the doctor.

E1

They also perceived benefits related to patients’ engagement in their care. From the point of view of physicians interviewed, mHealth apps could improve patients’ compliance with treatment and provide patients with reliable health-related information and education. The availability of targeted, easy-to-access information could help them manage their own health and adopt a healthy lifestyle:

Sometimes when we give advice, we don’t know what happens once they have gone home. If they have their application, it will support our advice about diet regimes, advice about care for certain chronic illnesses like diabetes... So, these are tools to help gain knowledge about their illness, to better understand the complications.

E20

### Patient Protection

Although GPs perceived benefits in terms of patient involvement and engagement, they also put forth a protective posture vis-à-vis their patients and the possible risks such apps could engender*.* Some of the risks mentioned were related to smartphone use and were falling within the lay representations of the technology:

- Addiction to smartphones:

It depends on the tool and depends on the usage of that tool. There are uses which become addictive, which become stressful, and harmful.

E11

- Dangers of cell phone radiation:

What does all the radiations do? (...) they say that telephones can cause brain tumors? (...) We are not 100% sure of their safety, so the person who is going to go around all the time with applications that link data, isn’t it potentially dangerous?

E3

- Distortion of ongoing relationships and cognitive changes:

The fact of having your telephone in hand all the time...it necessarily cuts off social interaction between people. And also, there are many cognitive changes when we are used to using apps from such a young age (...) we make less of an effort to remember things, less of an effort to search for information where it existed before, we have perhaps less critical distance from the sources we use (...) some sort of middle ground has to be found between using an application from time to time and without it severing all preexisting social ties.

E19

Another risk mentioned was related to the content of the apps that could provide irrelevant, unreliable, or nonevidence-based information:

There is always a risk that the information is not adapted to their case.

E18

From the point of view of the study participants, a certification performed by independent authorities, such as governmental agencies and health care professionals, could prevent the aforementioned risk:

I do not give advice to a patient if there is no scientific argument to back it up. I cannot suggest an app if it is not scientifically valid, or even independent, and if the protection of personal data is not guaranteed.

E2

They were also concerned about the security of the apps and had several fears about patients’ data privacy and security:

An app, proposed by a Lab [pharmaceutical industry]: NO! I would not trust it! [the risk would be] targeting, collecting patient data in their favor, to promote their products (...) because I think that the Lab collects patient data because the patients enter the data. To what end will they use this data? I am also there to protect my patients’ DATA, and I want to guide them in the use of applications that will not put their data protection at risk.

FGN-30.2

This protective posture was also fueled by the perception of another category of risks related to the management of patient care. From these GPs’ point of view, self-medication or self-management could lead to patient isolation, increase patients’ anxiety, or give patients a false sense of security:

The objective is not to collect data but to know how to analyze the data in a relevant and pertinent way. (...) it really must be standardized, otherwise there is a false sense of security: “I feel all alone.”

E8

Moreover, health apps are not seen as relevant tools for empowering patients because, as a participant said, “those who are going to get involved are patients who are already involved” (E17), whereas those who had little self-involvement in the management of their health will not become more empowered by mHealth apps:

We aren’t going to gain much. It will over-empower those who were already too much and...not change much.

E8

They also pointed to the risk of the standardization of the medical decision-making process based on patient monitoring with apps, which is opposed to a family practice. This standardization could lead to overmedicalization:

We can adapt these software programs all we like, I am not sure that we will ever be able to obtain the intuitive perspective that is the transcription of countless information that we have accumulated through experience and knowledge about our patients.

E20

They feared that apps might increase normative injunctions to achieve perfect health. In addition, they feared that patient autonomy requirements would put too much responsibility on patients and decrease their individual freedom:

Surveillance and self-management is all right but we shouldn’t be obsessed with all these settings. (...) There is too much information, I find, from the apps, the television, advice... (...) We have the right to cheat a bit, but I have the impression that we must live in a world that is more and more perfect.

E9

Finally, some of them suggested that apps could intensify social inequalities in health, being prescribed only to certain patients, depending on their income level, their level of digital literacy, and/or their linguistic ability:

What counts also, because I work in a difficult neighborhood, is applications that are adaptable–so, according to the languages the people speak, their level of literacy, because I am afraid that some apps are too specialized, and that deepens social inequalities of health!

FGN-30.1

### Doctor Protection

In response to the issues and challenges of the prescription of mHealth concerning medical practices, some GPs adopted an attitude that aims to protect their work and their professional position. They view the prescription of apps as an additional task. For some, giving related explanations would be too time consuming in the context of the current constrained duration of the medical encounter:

There are a lot of parameters to set up...For example, I tried the app for the pill: it really got on my nerves because by the time I found the right pill, I was running late! It isn’t manageable during a consultation! It blocks everything! For me, it should be a PLUS, and not take me more than 5 minutes, or...10 minutes; otherwise, I won’t be able to manage! (...) For an app to work, you have to enter the name, the age, and a certain number of other things that will take up too much of MY TIME! That is what I fear!

FGN-60

Apps would offer scattered information that might generate a great quantity questions from patients:

If the patient comes with more information (...) if he has read up, he gets off track. He has even more questions. (...) Raising questions is a very good thing because patients have to, of course, be informed...but at their level (...) during the consultation, there are often several reasons for the consultation, we have to think about performing cancer screening from the age of 50, we have to think about vaccinations... this is just adding yet another thing to think about (...) during the consultation, we just don’t have the time.

E10

Likewise, patients’ use of health apps might space out medical follow-up that would lessen the doctor’s acquaintance with his or her patient and increase the time necessary to know the important events that occurred in the meantime:

So they are more independent and self-reliant which is good, but when they come back to see us it will be a waste of time because what have they done during all this time? Why did they do this or that? The patients are less closely monitored.... So of course this will forcibly be more difficult to manage.

E18

The management of patient-gathered health data (such as patient-reported outcomes or patient data automatically generated by apps) will create extra work and increase the workload, especially because of the noninteroperability between the apps and the electronic medical record:

I REFUSE to be INUNDATED with “patient” data that arrive every evening and that I am supposed to have seen, with people who will come in and say “Have you seen my curves? What do you think about that?” That, that WOULD BE AWFUL!!! It would be the last straw! (...) That (the data flow) shouldn’t be an extra burden (...) So, I can imagine to what point the apps, if they provide a constant flow of information when the patient isn’t even here, would really make things difficult for me.

FGR-50

GPs are dubious about the value of patient-gathered health data compared with other clinical data to improve patient care and care planning and about the actionability of such data in the clinical realm:

I am bothered when people come and show me the results of their Freestyle app (...) I am lost with the averages in the result curves which I could care less about, and then we have to regulate the insulin...NO! the ONLY THING I AM INTERESTED IN is the morning blood sugar level!! I couldn’t care less about the rest!

FGR-50

They raised concerns of medical liability associated with remote monitoring:

The fear is that you prescribe mobile apps to all of your patients (including those with high blood pressure) and then, one night you are snug in bed and you receive a text message “Mr. So-and-So has a systolic of 200!!” [Laughter] ... you don’t sleep a wink all night!

FCN-40

Moreover, they emphasized that health apps should not be a substitute for diagnostic tasks:

The patient mustn’t go imagining that that (apps) will replace us. (...) it is just a digital tool and doesn’t have a global vision, a vision of everything and anything that could interfere with this or that... It must be a complement to what already exists; it must not replace the doctor’s point of view.

E15

mHealth apps are thought to be a disruptive technology that could alter care practices by reinforcing the purely technical aspects of medicine. Some GPs questioned the risk of transferring their role to the tools. Dehumanization of relationships is feared: mHealth apps could limit the specificities of general medical practice, such as negotiating with the patient’s follow-up plan and its adjustments, and might reduce interaction with patients and the tracking of emotions that are important to medical diagnosis and follow-up:

We might even be tempted to do that. (...) To delegate the work (...) (but) the empathy that we put into the explanation (of the illness), that is capital for the patient (...) we will have to learn to use that, but without forgetting our role, that does not discharge us from this role of explaining and sharing, especially (...) because that contact, when we no longer have it will be the moment when we become technicians and we will only have to apply algorithms.

E20

Another concern was the fear of increased drug prescriptions and health care expenses, especially if health apps are developed by the pharmaceutical industry:

There is an app that is officially provided by a patient organization, but in reality a lab (pharmaceutical industry) is behind it and financing it (...) The app is for the simple detection of AMD [age-related macular degeneration] (...) it is financed by the lab that supplies the products. The injection costs 800 euros to prevent AMD!

E4

That is why they were favorable to certification by independent authorities, such as governmental agencies and the health care professional community:

I would prescribe them (the apps) more readily if there were some authority that certified them and had the reputation of being independent...

E14

Finally, they fear the exploitation of people’s personal information, the commodification of patient data, and a new form of population monitoring:

The diffusion of the mass (of data) is something I fear much more. And that after that, we would treat certain sub-populations, in a statistical sense of the term, sub-populations with differentiated risk-management, that is a huge risk.

E8

## Discussion

### Principal Findings

On the basis of the perceptions and expectations of mHealth apps aimed toward patients of a sample of 36 French GPs, our study offers a typology that makes it possible to account for the different attitudes of GPs with regard to the recommendation of apps. Rather than discussing each category of attitudes, we analyzed the GPs’ fears, concerns, and tensions highlighted by the typology that can explain the resistance, on some of their parts, to recommend or prescribe mHealth apps to patients.

The first source of tension emerges in the approach of the doctor-patient relationship and the medical work of care management. mHealth apps are frequently presented in medical and public health literature as a means of empowering patients [25], and several GPs are sensible to this argument to prescribe mHealth. Several qualitative studies suggest that patients perceive mHealth apps as a means of self-monitoring and self-management of their health and an aid in gaining control and autonomy over their own health and health care [2]. Although the GPs interviewed approved patient engagement in their health care, they feared risks that did not seem to have been disclosed by previous studies. They pinpointed risks related to doctor-patient relationships (dehumanization), patient follow-up (isolation, anxiety, self-medication, false sense of security, numerical standards for normalcy, and disappearance of doctor mediation in the interpretation of data), and medical practice (substitution for medical diagnosis, overmedicalization, standardization of follow-up and prescription, and reduction of professional autonomy). Finally, like their “skeptical” German colleagues [18], the GPs interviewed stressed security, privacy, and confidentiality concerns about patient-generated data.

mHealth can generate tensions if the tool provides patient information that the GP does not need for his or her expertise or if the flow of information disrupts medical work, reduces valuable medical time, or increases the workload. This is consistent with a previous study [18]. To manage care and follow-up, GPs require different information about their patients. Therefore, mHealth apps can support medical practice in generating patient data [1]. However, as shown by previous studies, doctors “feel more comfortable about data that they have collected themselves about the patient (*...*) rather than those collected by patients themselves on their own initiative” [26]. To consolidate their expertise (medical decision making and plan care), they expect data “valence of actionability” [27], which refers to patient-generated data integration in clinics and the physician’s responsibility in subsequent medical interventions.

The GPs interviewed stressed the lack of guidelines for patients’ use of mHealth apps and the need for independent certification to ensure quality and trustworthiness of the apps before recommending them, as suggested by previous studies [14,15]. Certification of mHealth apps could be a way to increase GPs’ confidence in mHealth apps and promote the digital engagement of physicians. However, the huge production of new mHealth apps renders certification tasks more complex [28].

Our typology underlines tensions in GP’s medical work that would be induced by patients’ use of mHealth apps. However, these tensions could be experienced by GPs differently, depending on national health care contexts and considering the various patient requirements and expectations that may differ according to the diverse organizations of health care systems in other countries. For instance, some mHealth apps may be alternatives to medical consultation in developing countries where access to health care is restricted or in industrialized countries where care costs are substantial and for patients living far from health care services. On the other hand, a solidarity-based public health insurance system, such as the French system that covers 70% to 100% of primary care costs, does not favor patient self-care. As a result, prescribing mHealth apps is more likely to be perceived by GPs as a supplementary workload. However, the globalized mHealth technologies market proposes the use of mHealth apps without distinguishing between the different expectations of each relevant actor in each country.

Finally, transversal to these different attitudes, in our study, most GPs consider the smartphone to be an integral part of daily lives of many people. Not all physicians interviewed were entirely convinced that mHealth apps have the potential to revolutionize the management of disease. However, they regarded the introduction of mobile apps as an inevitable development in the field of medicine and one that could be leveraged to change patients’ behavior.

As we have emphasized, each GP interviewed could have an ambivalent discourse with regard to specific aspects of the prescription of mHealth apps or patients’ use of apps. Some sociological approaches propose a nuanced understanding of users’ attitudes toward digital health [29]. Rather than the classical opposition found in the literature between “resistance to” [30-33] and “acceptance of” [34-37] digital health technologies, Marent et al [29] prefer to use the concept of ambivalence as a “means of facilitating and capturing contradictions and tensions in accounts of digital health” [29]. According to these authors, “ambivalence is the simultaneous experience of two (ambi) opposing orientations or values (valences)” that “indicates an oscillation or tension between opposite poles of feeling and thinking,” at an individual or collective level. In the case of the physicians interviewed, we hypothesize that this ambivalence is generated by a tension between the desire to see patients’ commitment to care progress and the fear of seeing their medical authority challenged [26]. To alleviate this tension, some GPs interviewed adopted a paternalistic stance [26] by highlighting their medical responsibility to protect patients and medical ethics [38].

From a daily work perspective, our findings could help GPs to identify the category of attitudes with regard to the recommendation of apps in which they fall and to make them aware of their ambivalence. They could then anticipate strategies to lessen their personal constraints on practices such as task delegation, personal training, adequate medical record software choice, production of information devices for patients, and identification of apps that match their values and expectations. Subsequently, they could slowly but steadily start prescribing mHealth apps to targeted patients at targeted times.

### Limitations

The main limitation of this study is the high rate of nonrespondents (72/90, 80%). Moreover, the recruitment process led to a high number (30/36) of GPs involved in medical training. This overrepresentation may be a bias because respondents, especially physician trainers, could be aware of mHealth and more willing to participate in the study. It is possible that the nonrespondents were not interested in mHealth and, therefore, were even less concerned about their prescription.

However, the “thick description” [39] of the various GPs’ postures and their latent meanings revealed by sociological analysis make our typology transferable to other national contexts [39].

### Recommendations for Implementation

As GPs are leading providers of health information and advice to patients, a deep understanding of both expectations and fears of GPs is essential to motivate them to recommend mHealth apps to their patients. To this end, there is a need to provide appropriate education and training to enhance the digital skills of GPs [15,40]. Our findings show that certification of the apps by an independent authority should be encouraged to reassure physicians about ethical and data security issues [14,15,28]. Our findings also highlight the need to overcome technical issues such as the interoperability between data collection and medical records to limit the disruption of medical work by the data flow. It could be useful to reorganize the medical work, with consultation devoted both to the prescription of apps and the interpretation of the patient-generated data, to lift economic barriers and to preserve personalized management of patient care.

We can anticipate that the availability and assistance of self-monitoring apps in the context of the response to the COVID-19 epidemic to follow infected patients [41,42] could impact the willingness of GPs to prescribe mHealth apps to their patients.

### Conclusions

The results obtained using a sociological analysis clearly reveal the tight imbrication of social and cultural factors underlying the attitudes of GPs toward prescriptions of mHealth apps. Our analysis could be informative for medical teachers, public health decision makers, and all professionals grappling with questions of integrating mHealth in medical practices.

## Appendix

Multimedia Appendix 1 Illustrative quotes.

## Abbreviations

FG: focus group
GP: general practitioner
mHealth: mobile health
