# Supplementary material for: Attitudes of General Practitioners Toward Prescription of Mobile Health Apps: Qualitative Study
Source: JMIR Mhealth Uhealth. 2021 Mar 4;9(3):e21795. doi: 10.2196/21795 (PMC7974757; doi:10.2196/21795)
Supplement: Multimedia Appendix 1 [file mhealth_v9i3e21795_app1.docx]

**Multimedia Appendix 1.** Illustrative quotes.

| *Theme: GPs’ willingness: apps as additional resource to medical work* | |
| --- | --- |
| **Subthemes** | **Illustrative quotes** |
| Familiarity with the tool | *E19: One that is very often recommended for chronic lumbago is the application Activ’Dos. I know that one well because I have used it myself. It is rather well done. (…) I tested it on myself, so I advise it for my patients and show them how to use it (…) and actually showing them the application is very different from writing the name down on a post-it note because they can see for their own eyes what it can do… (…) I think that there is truly a positive impact by showing them what it looks like.* |
| Reliability and objectivity of measures | *E8: Objectivity, increased precision on dynamic measurements. Because it is the repetition of measurements that will yield the most pertinent average figures.* |
| History of measurements | *E15: (blood pressure measurements) an application would be much better. I give them papers with charts to fill in, then they bring me their charts…which is just fine! I scan them and put them in the files, but if I had that on the internet, it would be…much simpler.* |
| Better monitoring of the patients | *E10: It should be a tool that facilitates the transmission of information from him to me. (…) It would be a bit like having the nurse visit the patient every day (…) with an eye on the patient’s state of health on a daily basis. I think that would be useful.* |
| Remote decision-making and follow-up | *E7: The insulin-dependent diabetic patient must be self-reliant. So, we can find applications that would be doubly interesting; 1) for the patients, 2) through the communication of data because there is a stake in using mobile apps. The stake is double. It is 1) managing patient data and facilitating and aiding patients’ decision, notably for diabetes, 2) the major interest is the communication of data (…) so the interest is double for the diabetic, (…) there is no need to go anywhere. All of the data are transferred to the doctor. He has, for example, a week of (blood sugar) curves and can remotely (…) adapt the insulin and the dosage and the therapeutic measures for the diabetic.* |
| Self-monitoring and education | *E8: Where it can be a little useful, perhaps, is for the follow-up on the prescription of physical activity (…) in the care for metabolic syndromes, among others, and the prevention of osteoporosis, etc. (If) we have a report from the program about the physical adaptation of the patient, that…that can be a plus, because that will allow the measurement of something that pertains to a non-pharmacological treatment of the measures of impact…that, yes!* |
| Complementary tools to medical practice | *E16: (we can imagine) a program that explains self-reeducation therapy of the knee after a prosthesis, but as a complement to physical therapy with a therapist, so that people would have a program where they see what kind of movements they should do at home to complement what the physical therapist does.* |
| *Theme: GPs’ skepticism or resistance* | |
| **Subthemes** | **Illustrative quotes** |
| Apps should not be a substitute to diagnostic task. | *E1: (the apps) this would be to help the doctor on a daily basis, or to help the patient in the care and management of his illness, but certainly not to make medical diagnoses or to provide pseudo-diagnoses (…) in the place of the doctor.*  *E15: The patient mustn’t go imagining that that (apps) will replace us. (…) it is just a digital tool and doesn’t have a global vision, a vision of everything and anything that could interfere with this or that… It must be a complement to what already exists, it must not replace the doctor’s point of view.* |
| Too time consuming | *FCN-60: (a first trial with a patient) there are a lot of parameters to set up…For example, I tried the app for the pill: it really got on my nerves because by the time I found the right pill, I was running late! It isn’t manageable during a consultation! It blocks everything! For me, it should be a PLUS, and not take me more than 5 minutes, or…10 minutes, otherwise, I won’t be able to manage! (…) For an app to work, you have to enter the name, the age, and a certain number of other things that will take up too much of MY TIME! That is what I fear!*  *E10: if the patient comes with more information (…) if he has read up, he gets off track. He has even more questions. (…) Raising questions is a very good thing because patients have to, of course, be informed…but at their level (…) during the consultation, there are often several reasons for the consultation, we have to think about performing cancer screening from the age of 50, we have to think about vaccinations… this is just adding yet another thing to think about (…) during the consultation, we just don’t have the time.*  *E18: So they are more independent and self-reliant which is good, but when they come back to see us it will be a waste of time because what have they done during all this time, why did they do this or that? The patients are less closely monitored….so of course this will forcibly be more difficult to manage.* |
| Additional task | *E4: (on push messages from the app) So, when am I going to do that? On top of the administrative work? (...) we also have to take the time to read and interpret results.*  *FCR-50: I REFUSE to be INUNDATED with “patient” data that arrive every evening and that I am supposed to have seen, with people who will come in and say “Have you seen by curves? What do you think about that?. That, that WOULD BE AWFUL!!! It would be the last straw! (…) That (the data flow) shouldn’t be an extra burden (…) So, I can imagine to what point the apps, if they provide a constant flow of information when the patient isn’t even here, would really make things difficult for me.* |
| Requiring independent certification | *E14: I would prescribe them (the apps) more readily if there were some authority that certified them and had the reputation of being independent…* |
| Requiring clinical validation | *E2: I do not give advice to a patient if there is not a scientific argument to back it up. I cannot suggest an app if it is not scientifically valid, or even independent, and that the protection of personal data be guaranteed.* |
| Risk of increasing drug prescriptions and healthcare expenses | *E4: There is an app that is officially provided by a patient organization, but in reality a lab (pharmaceutical industry) is behind it and financing it (…) The app is for the simple detection of DMLA (…) it is financed by the lab that supplies the products. The injection costs 800 euros to prevent DMLA!* |
| Patients’ data protection | *FCN-30: an application about data collection, in mode “Weight, Height, ECG, medication, habits, etc.”, I would not advise such a thing without being sure about data protection.* |
| Medical liability associated with remote monitoring | *FCN-40: The fear is that you prescribe mobile apps to all of your patients (including those with high blood pressure) and then, one night you are snug in bed and you receive a text message “Mr. So-and-So has a systolic of 200!!” ….[laughter]… you don’t sleep a wink all night!*  *FCR-60: It is also about the problem of the responsibility when faced with the data that arrives.* |
| No value to improve care planning | *FCR-50: I am bothered when people come and show me the results of their Freestyle app (…) I am lost with the averages in the result curves which I could care less about, and then we have to regulate the insulin…NO! the ONLY THING I AM INTERESTED IN is the morning blood sugar level!! I couldn’t care less about the rest! So sometimes, it disturbs the relationship with the patient because you find yourself managing a flow of information among which there isn’t much of interest for you, so, it adds a constraint to the consultation: you have to listen to and look at all of the result curves the patient wants to show you, whereas that has little influence over your decision.* |
| *Theme: High value of the apps for patient* | |
| **Subthemes** | **Illustrative quotes** |
| Smartphone is an everyday tool | *E1: they are all equipped to have apps (…) at least the telephone is the thing they never forget at the consultation (laughter). The blood sugar notebook…the paper notebook (…) they forget that all the time, but the telephone is something they never forget.* |
| Improving patients’ compliance | *E3: Well, if there are reminders, for example a reminder to take the pill, that is good because it prevents forgetfulness. Very simple and precise things, that is good because the forgetful patients are often absent-minded as well.* |
| Assisting in therapeutic education | *E20: the advantage is indeed having information at hand that can be repeated. We can provide the initial information and then they will have something that is perhaps more precise and complete and that adds on to what we have said. Sometimes when we give advice, we don’t know what happens once they have gone home. If they have their application, it will support our advice about diet regimes, advice about care for certain chronic illnesses like diabetes…So, these are tools to help gain knowledge about their illness, to better understand the complications (…). Educating the patient is very important.* |
| Promote an healthy lifestyle | *FCR-50: It can be a supportive tool for change…a strong lever for change for the patients. It makes me think of….well, it is outside the medical field, but I see how an app like WAG can provide examples… (…) it is an application to help change habits in terms of green consumption and planet-friendly behavior. Nothing to do with medicine, but I tried it out personally and I think that it is surely something to do with patients.* |
| Availability of information | *E19: Having (information) on the telephone isn’t any more interesting than having a brochure. Well, but it is information that we have on hand, that we read anywhere (…) I’m in the train…and why not, I’ll do a little quiz about blood sugar!* |
| Reliability of information | *E19: (the apps) it provides me with a tool to give them reliable information when it is really trustworthy, there is no guarantee that there are less risks of getting false information, finding erroneous sources about illnesses, about treatments, etc.* |
| Increasing patient engagement | *E1: Even just filling in the information, or keeping to filling in information in the application will help them with their illness because they will be actors of their own health (…) they will be more involved and that will be better for their care (…) it will help with their follow-up. I think that they will be in better health just because they oblige themselves to do something for themselves.*  *E19: I like the idea of giving our patients tools to take charge of themselves, that we enable them to be agents of their own healing, of their healthcare, and the fact of having an application with exercises and images, etc., it is better than writing out a prescription and then re-writing a prescription for physical therapy whereas…. It works, but it is better if it is complemented with self-care management exercises. It is the idea that patients can obtain information on their own and know how to do things, to become actors on their illness.* |
| Increasing patients’ empowerment | *E2: Providing an application is entering into the daily life of the patient, it is putting them in a situation of self-management, it is trusting them, it also teaches them that they should not be our captives (…) To my mind, the prescription of applications can favor the relationship in my practice rather than diminish the quality of that relationship. Because, in addition to being a shared decision, it is a shared prescription. (…) It can be included in the panel of tools, the patients like tools, I use them, they can refuse it (…) I leave the patients choose freely the resources that they want.* |
| *Theme: less value of the apps and risks for patient* | |
| **Subthemes** | **Illustrative quotes** |
| Addiction | *E11: It depends on the tool and it depends on the usage of that tool. There are uses which become addictive, which become stressful, and harmful.* |
| Dangers of cell phone radiation | *E3: What does all the radiation do? (…) they say that telephones can cause brain tumors? (…) We are not 100% sure of their safety, so the person who is going to go around all the time with applications that link data, isn’t it potentially dangerous?* |
| Distortion of ongoing relationships, and cognitive changes | *E19: The fact of having your telephone in hand all the time…it necessarily cut off social interaction between people. And also, there are many cognitive changes when we are used to using apps from such a young age (…) we make less of an effort to remember things, less of an effort to search for information where it existed before, we have perhaps less critical distance from the sources we use (…) some sort of middle ground has to be found between using an application from time to time and without it severing all preexisting social ties.* |
| Irrelevant or unreliable | *E18: The risks arise when they glean information on their own. There is always a risk that the information is not adapted to their case.* |
| Patients’ data privacy and security | *E11: Today, there is no quality label and no guarantee about the security of data (…) While we pay these applications because they provide something (I do agree) but we should keep the data that they produce, that is where there is a significant void and today, personally, I cannot advise my patients about a certain number of applications in which the data is not protected (…) It must be ethical, the government should take a stand about that, clearly (…) about regulations concerning this data.*  *FCN-30.2: An app, proposed by a Lab (pharmaceutical industry): NO! I would not trust it! (the risk would be) targeting, collecting patient data in their favor, to promote their products (…) because I think that the Lab collects patient data because the patients enter the data. To what end will they use this data? I am also there to protect my patients’ DATA, and I want to guide them in the use of applications that will not put their data protection at risk.* |
| Self-medication | *E8: I am suspicious about the self-medication that this might lead to (…) Many patients, when we have them take measurements themselves say “my blood pressure was fine, I stopped the treatment”…* |
| Patient isolation | *E10: The risk is that he confront his illness isolated and alone, because he has an application for that illness…and he thinks to himself “the good doctor gave me this app, I am not going to go bother him for one question.”* |
| Increasing patient’s anxiety | *E1: The fact to self-manage (…) for example their blood sugar (…) Their blood sugar level is a little high, so they keep on checking their blood sugar, and they get all anxious over nothing, and they will check their blood sugar 40 times which will get them even more worried because they don’t know how to handle that. And the application, it isn’t there to take over the doctor’s role.*  *E19: If there is a notification every 15 minutes because the application detected whatever parameter (slight laughter)… that can have completely harmful effects on people and keep them from living life to the fullest and letting go. It can have completely perverse, yes, perverse effects.* |
| False sense of security | *E8: The biggest risk that I see is the illusion of surveillance that does not lead to anything because we lose scope of the objective. The objective is not to collect data but to know how to analyze the data in a relevant and pertinent way. (…) it really must be standardized, otherwise there is a false sense of security: “I feel all alone”.* |
| Standardization of the medical decision-making and over-medicalization | *E19: The art of medicine is to know how to adapt to the individual situation of each patient and it is true that we cannot oblige people to follow a diet, to note down their sugar intake, etc. There are truly situations that are very, very diverse and varied, and standardizing that in an application, I think that will miss the point, and people won’t use it.*  *E20: The risk of all these standardized things is indeed over-medicalization and we can see that very clearly at the GP level, we adjust and moderate…for example, prescribing a treatment of hypertensives…(…) sometimes the good common sense of a good old GP who knows the family well, who knows the patient well, will adjust what would have been an aggressive therapy …(…) so, aren’t these types of tools going to apply extremely standardized and less adapted care strategies on each patient? Because, we can adapt these software programs all we like, I am not sure that we will ever be able to obtain the intuitive perspective that is the transcription of countless information that we have accumulated through experience and knowledge about our patients.* |
| Normative injunctions | *E9: People have to be able to live as well, without paying too much attention to surveillance (…) therein lies the danger of the weight, the number of kilometers we walked in a day, our body temperature…It is all too standardized. We have the right to cheat a little with our sugar intake. Surveilling and self-management is alright but we shouldn’t be obsessed with all these settings. (…) There is too much information, I find, from the apps, the television, advice… (…) we have the right to cheat a bit, but I have the impression that we must live in a world that is more and more perfect.*  *E20: My worry, is that this harms the way the patients see themselves (…) where everything would boil down to control. We have to control our heart rate, control our blood pressure (…) and so, we fabricate professional patients (…) That is what scares me,… that he spends too much time on these gadgets that beep all the time. Not a second to think about anything other than his illness (…) I think that we are already far too intrusive in peoples’ lives (…) Well, it is good to be healthy but if that takes away from living, life isn’t worth living.* |
| Dehumanization of the relationship | *E20: We should not transfer our role onto these tools (…) and let the patient with a text message about their illness. We will, effectively, save time, we wouldn’t have to spend an hour explaining what severe illness is, we might even be tempted to do that. (…) to delegate the work (…) (but) the empathy that we put into the explanation (of the illness), that is capital for the patient (…) we will have to learn to use that, but without forgetting our role, that does not discharge us from this role of explaining and sharing, especially (…) because that contact, when we no longer have it will be the moment when we become technicians and we will only have to apply algorithms.* |
| Not relevant tools for empowering patients | *E8: We aren’t going to gain much. It will over-empower those who were already too much and…not change much.* |
| Increasing social inequalities of health | *FCN-30.1: What counts also, because I work in a difficult neighborhood, is applications that are adaptable – so, according to the languages the people speak, their level of literacy, because I am afraid that some apps are too specialized, and that deepens social inequalities of health! (…) I would find it crazy that certain (social) classes could have this type of information! And there are some people who don’t own a smartphone!* |
| Population monitoring | *E8: The diffusion of the mass (of data) is something I fear much more. And that after that, we would treat certain sub-populations, in a statistical sense of the term, sub-populations with differentiated risk-management, that is a huge risk.* |
